# *Clonorchis sinensis* antigens alter hepatic macrophage polarization *in vitro* and *in vivo*

**DOI:** 10.1371/journal.pntd.0005614

**Published:** 2017-05-24

**Authors:** Eun-Min Kim, You Shine Kwak, Myung-Hee YI, Ju Yeong Kim, Woon-Mok Sohn, Tai-Soon Yong

**Affiliations:** 1 Department of Environmental Medical Biology and Arthropods of Medical Importance Resource Research Bank, Institute of Tropical Medicine, Yonsei University College of Medicine, Seoul, Korea; 2 Department of Parasitology and Tropical Medicine, and Institute of Health Sciences, Gyeongsang National University School of Medicine, Jinju, Korea; Seoul National University College of Medicine, REPUBLIC OF KOREA

## Abstract

*Clonorchis sinensis* infection elicits hepatic inflammation, which can lead to cholangitis, periductal hepatic fibrosis, liver cirrhosis, and even cholangiocarcinoma. Hepatic macrophages are an intrinsic element of both innate and acquired immunity. This study was conducted to demonstrate the dynamics of hepatic macrophage polarization during *C*. *sinensis* infection in mice and to identify factors regulating this polarization. Treatment of hepatic macrophages isolated from normal mice with *C*. *sinensis* excretory/secretory products (ESPs) resulted in the preferential generation of classically activated hepatic macrophages (M1 macrophages) and the production of pro-inflammatory cytokines. Additionally, cells stimulated with *C*. *sinensis* ESPs exhibited changes in cellular morphology. During the early stages of *C*. *sinensis* infection, hepatic macrophages preferentially differentiated into M1 macrophages; however, during the *C*. *sinensis* mature worm stage, when eggs are released, there were significant increases in the abundance of both M1 macrophages and alternatively activated hepatic macrophages (M2 macrophages). Moreover, there was a further increase in the M2 macrophage count during the fibrotic and cirrhotic stage of infection. Notably, this fibrotic and cirrhotic stage promoted a strong increase in the proportion of Arg-1-producing macrophages (M2 phenotype), which were associated with fibrosis and tissue repair in the liver. Our results suggest that the dynamic polarization of hepatic macrophages as *C*. *sinensis* infection progresses is related to the histological lesions present in liver tissue. Hepatic macrophages thus play an important role in local immunity during *C*. *sinensis* infection.

## Author summary

Infection with *Clonorchis sinensis* is a major public health problem in Asia, resulting in loss of liver function and chronic liver diseases, including cancers. However, to the best of our knowledge, the immune response to fluke infection in the liver has not been systematically investigated. Here, we demonstrated that *C*. *sinensis* infection triggered a shift in the characteristics of macrophages, the primary cells associated with host immunity, throughout the course of the worm’s life cycle. Eventually, the increased number of M2 macrophages may result in fibrosis and the remodeling of bile ducts within the liver. Our findings suggest that, while the infection initially led to changes in the immune response that facilitated *C*. *sinensis* survival, ongoing infection may also reduce the severity of disruption of liver function. Hepatic macrophages activated during *C*. *sinensis* infection may not only be operating in histological lesions around the bile duct, but may also play a role in local immunity.

## Introduction

*Clonorchis sinensis* infection represents a major public health problem in Asia, with approximately 30 million people infected, of which 1.3 million reside in Korea [1, 2]. *C*. *sinensis* infection causes several pathological alterations in the bile ducts, including hyperplasia of the mucosa, dilatation of the bile duct, and fibrosis in the region surrounding the bile duct. Chronic and heavy infections can cause cholangitis or abscess formation, pancreatitis, gallstone formation, and cholangiocarcinoma [1–3].

*C*. *sinensis* infection is distinguished by an adaptive immune response related to a Th2 phenotype [4]. Although Th2-type cytokines and other host-protective immune responses attack parasites, the parasites are in turn able to control the host immune response to maintain survival, consequently leading to chronic infection [5]. However, despite the presence of *C*. *sinensis* in the bile duct, only few reports have been published regarding the local immune response to the parasite within the liver and bile duct.

Macrophages can be classified into two subpopulations: classically activated macrophages (M1 macrophages) and alternatively activated macrophages (M2 macrophages). M1 macrophages are distinguished by the expression of high levels of inducible nitric oxide synthase (iNOS), tumor necrosis factor (TNF)-α, CD16/32, and chemokines such as chemokine (C-X-C) motif ligand (CXCL) 9, CXCL10, and CXCL11; these macrophages are generated in response to pro-inflammatory stimuli such as lipopolysaccharide (LPS) [6–10]. Conversely, M2 macrophages are distinguished by their high expression of arginase-1 (Arg-1), interleukin (IL)-10, CD206, and chemokines such as C-C motif chemokine ligand (CCL) 2 and CCL22, and develop in response to inflammatory stimuli such as IL-4 [11–13]. However, the specific contributions of hepatic macrophage proliferation to infection control and/or the restoration of tissue homeostasis remain unclear.

Macrophages differentiate into M1 and M2 functionalities following infection with microorganisms or parasites [10]. In this regard, the complex interaction between excretory/secretory proteins (ESPs) from *Fasciola hepatica* and peritoneal macrophages is crucial for the establishment of this parasite in mice. Moreover, treatment with *F*. *hepatica* ESPs has been shown to induce alternatively activated macrophages in experimental models [11, 13]. Among hepatic macrophages, resident macrophages termed Kupffer cells evoke an important element of innate immunity and display an early, rapid response to dangerous stimuli [9, 14]. Another group of hepatic macrophages appears to be derived from circulating monocytes. In particular, bone marrow-derived monocytes migrate into various organs, where they are converted into organ-specific macrophages [15–17]. Upon activation, these different classes of hepatic macrophages release products such as cytokines, chemokines, nitric oxide (NO), and reactive oxygen species (ROS). Thus, hepatic macrophages are involved in the liver’s immune response to infection and other stressors [18, 19].

To the best of our knowledge, the dynamics of hepatic macrophage polarization during *C*. *sinensis* infection have not been reported. In this study, we analyzed M1 and M2 phenotypes to identify the presence and degree of macrophage polarization in the liver upon infection with *C*. *sinensis*. Possible roles for *C*. *sinensis* ESPs in hepatic macrophage polarization were also investigated. These results may provide insights into the development of methods to better control hepatic fibrosis in clonorchiasis.

## Materials and methods

### Ethics statement

The animal experiment protocol was approved and reviewed by the Institutional Animal Care and Use Committee (IACUC) of Yonsei University Health System, Seoul, Korea (approval no. 2015–0339) and followed the National Institutes of Health (NIH) guidelines for the care and use of laboratory animals (NIH publication no. 85–23, 1985, revised 1996). The animal facility was certified by the Ministry of Food and Drug Administration and by the Ministry of Education, Science, and Technology (LNL08-402).

Animal experiments were carried out in animal biosafety level-3 (ABL-3) facilities in accordance with ABL-3 standard managing practices. *C*. *sinensis* metacercariae were obtained from the freshwater fish *Pseudorasbora parva*, caught at Sancheong, Gyeongsangbuk-do, Korea.

### Experimental groups and infection of mice with metacercariae of *C*. *sinensis*

Male, 5-week-old BALB/c mice were purchased from Orientbio (Seongnam, Korea). The animals were randomly divided into uninfected or infected groups (n = 10 for each group). Mice in the infected group were orally infected with 30 metacercariae. The experiment was designed to focus on the juvenile worm and egg-emission stages of infection. As such, the first group of mice was studied during the early infection stage, 1 to 2 weeks post-infection. The second and third groups were studied 7 and 8 weeks post-infection, respectively, representing the egg-production stage of mature worms, and the fourth group was studied 10, 12, and 16 weeks post-infection, during the fibrotic and cirrhotic stage. Mice were euthanized under deep anesthesia with ethyl ether and each experiment was performed in duplicate.

### Preparation of ESPs from *C*. *sinensis*

Male, 5-week-old Sprague-Dawley (SD) rats (Orientbio Inc.) were individually infected with 50 metacercariae of *C*. *sinensis*. At 8 weeks post-infection, the rats were euthanized under deep anesthesia with ethyl ether, before adult worms were isolated from the bile ducts, washed five times with phosphate-buffered saline (PBS) containing 100 U/ml penicillin/streptomycin (Sigma Aldrich, St. Louis, MO, USA), then incubated for 24 h at 37°C with 5% CO_2_. After incubation, the medium was centrifuged for 10 min at 1,000 × *g* to remove the worms and cellular debris. The supernatant was then centrifuged for a further 10 min at 18,000 × *g* before being filtered with a syringe-driven 0.45 μm filter. The concentration of protein was measured using Bradford assay reagent (Thermo Fisher Scientific, Waltham, MA, USA).

### Histopathology

The livers of five mice from each group were removed, and five transverse sections were produced per lobe. The liver sections were then fixed in 10% phosphate-buffered neutral formalin. The specimens were embedded in paraffin and stained with hematoxylin and eosin (H&E) and periodic acid-Schiff (PAS) to observe any histopathological lesions.

### Hepatic macrophage preparation

Nonparenchymal liver cells were isolated via the pronase-collagenase method, as described previously [18]. Briefly, portal veins were intubated with a plastic catheter (2 mm diameter), and livers were perfused *in situ* with 10 ml PBS containing 0.1% mixed type IV collagenase (10 ml/min) at 37°C in a nonrecirculating design to remove red blood cells. Livers were then transferred to 35 mm culture dishes and minced into small pieces. The liver tissues were dispersed in 10 ml Roswell Park Memorial Institute 1640 medium (RPMI 1640; Hyclone, Logan UT, USA) containing 0.1% type IV collagenase at 37°C for 30 min, then mixed gently with a graduated pipette for 10 min. After digestion, the liver homogenates were filtered through a 74-μm stainless steel wire mesh to remove undigested tissue, and the resulting cell suspensions were centrifuged at 300 × *g* (5810R; Eppendorf, Hamburg, Germany) for 5 min at 4°C. The aqueous phase was discarded, and the cell sediment was suspended in RPMI 1640, transferred into a new 10-ml centrifuge tube, and centrifuged at 300 × *g* for 5 min at 4°C. Again, the aqueous phase was discarded, and the resulting cell sediment was seeded into a T-75 flask at a density of 1–3 × 10^8^ cells/well in Dulbecco’s modified Eagle’s medium (DMEM; Hyclone) supplemented with 10% fetal bovine serum (FBS; Hyclone) and 100 U/ml penicillin/streptomycin (Sigma-Aldrich). Cells were then incubated for 2 h in a 5%-CO_2_ atmosphere at 37°C. Nonadherent cells were removed from the dish; the adherent cells were considered hepatic macrophages.

### *In vitro* treatment of hepatic macrophages and cytokine quantification

Hepatic macrophages were purified from normal mice and then cultured in medium containing either PBS, 10 μg/mL ESPs, 100 ng/mL LPS (Sigma Aldrich), or 10 ng/ml IL-4 (Peprotech Inc., Rocky Hill, NJ, USA) for 48 h at 37°C. The macrophages treated with LPS or IL-4 were regarded as positive controls for M1 and M2 macrophages, respectively.

Following *in vitro* treatment, macrophages were stimulated with *C*. *sinensis* ESPs (10 μg/mL), and supernatants were harvested after 48 h. TNF-α, IL-6, and IL-13 levels were determined by enzyme-linked immunosorbent assays (ELISAs), according to the manufacturer’s instructions (Peprotech Inc.).

### Flow cytometry

Single-cell suspensions were adjusted to 1 × 10^6^ cells/100 μL PBS containing 1% FBS. To analyze hepatic macrophage purity, cells were incubated with a fluorescein isothiocyanate (FITC)-conjugated antibody specific for mouse F4/80, a macrophage/microglial marker, and an APC-conjugated antibody specific for mouse CD11b (BioLegend, San Diego, CA, USA). For M1 and M2 surface marker analysis, cells were incubated for 1 h at 4°C with either PE-conjugated or Alexa 647-conjugated antibodies specific for mouse CD16/32 or CD206 (BioLegend; 10 μg/mL each), respectively, then washed with PBS. The hepatic macrophages were then fixed with 1% paraformaldehyde/PBS and analyzed using a BD FACSCalibur Flow Cytometer (BD Biosciences, San Jose, CA, USA). Results were analyzed using FlowJo software (BD Biosciences).

### Confocal microscopy

For microscopic examination, the hepatic macrophages were washed three times with cold PBS, fixed with 2% paraformaldehyde in PBS for 30 min, then permeabilized with 0.2% (w/v) Triton X-100 in PBS for 5 min. Hepatic macrophages were then blocked with 0.5% bovine serum albumin in PBS for 1 h before being incubated with an Alexa-Fluor 488 Avidin-labeled antibody detecting F4/80, a macrophage marker (Invitrogen, Carlsbad, CA, USA). Nuclei were visualized by staining with 4',6-diamidino-2-phenylindole (DAPI), and cells were observed under an LSM PASCAL confocal laser scanning microscope (Carl Zeiss, Oberkochen, Germany).

### Immunohistochemistry

For immunohistochemistry analyses, heat-induced epitopes in liver sections were retrieved by incubation with proteinase K at 100°C in a water bath for 1 h, followed by incubation with an anti-rabbit F4/80 antibody (1:50 dilution; LifeSpan Biosciences, Seattle, WA, USA) for 24 h at 4°C in a humidified chamber. After washing with PBS, the slides were incubated with an anti-mouse secondary antibody (LifeSpan Biosciences) for 1 h at room temperature, and visualized using diaminobenzidine and hematoxylin as the counter stain. After detection by F4/80 staining, hepatic macrophages were semi-quantified by viewing 10 fields at 100× magnification, using an Olympus light microscope (Tokyo, Japan).

### Real-time polymerase chain reaction (PCR) analysis

Total RNA was isolated from hepatic macrophages, using TRIzol reagent (Invitrogen) and converted to cDNA, using a Reverse Transcription Kit according to the manufacturer’s instructions (Fermantas Life Sciences, Waltham, MA, USA). Quantitative real-time PCR was then conducted using SYBR Green Master (Rox) reagents (Roche Diagnostics, Basel, Switzerland) and a 7300 Real-time PCR machine (Applied Biosystems, CA, USA). The reaction conditions were as reported previously [20]: stage 1, 50°C for 2 min; stage 2, 95°C for 10 min; stage 3, 45 cycles of 95°C for 15 s and 60°C for 1 min, followed by a melting curve analysis process. Fold changes in gene expression were calculated using the 2^−ΔΔCt^ method. The sequences of the primer pairs used for these analyses were as follows: *Tnfa* (forward) 5′-CATCTTCTCAAAATTCGAGTGACAA-3′ and (reverse) 5′-TGGGAGTAGACAAGGTACAACCC-3′ [20]; *Il10* (forward) 5′-ACTTTAAGGGTTACTTGGGTTGC-3′ and (reverse) 5′-ATTTTCACAGGGGAGAAATCG-3′; *Cxcl9* (forward) 5′-TCTCGGACTTCACTCCAACACA-3′ and (reverse) 5′-ACTCCACACTGCTGGAGGAAGA-3′; *Ccl2* (forward) 5′-AAGCCAGCTCTCTCTTCCTCCA-3′ and (reverse) 5′-AAGCCAGCTCTCTCTTCCTCCA-3′; *Nos2* (encoding the iNOS protein) (forward) 5′-GCCACCAACAATGGCAACA-3′ and (reverse) 5′-CGTACCGGATGAGCTGTGAATT-3′; *Arg1* (forward) 5′-CAGAAGAATGGAAGAGTCAG-3′ and (reverse) 5′-CAGATATGCAGGGAGTCACC-3′ [20]; and *Gapdh* (forward) 5′-GGTGAAGGTCGGTGTGAACG-3′ and (reverse) 5′-ACCATGTAGTTGAGGTCAATGAAGG-3′.

### Statistical analysis

The values in each graph represent the means ± standard deviations (SDs) of the results obtained from independent experiments. Data were analyzed by Student’s t-tests, and *p*-values of less than 0.05 were considered statistically significant.

## Results

### Effect of *C*. *sinensis* ESPs on hepatic macrophage polarization and morphology *in vitro*

**Fig 1:**
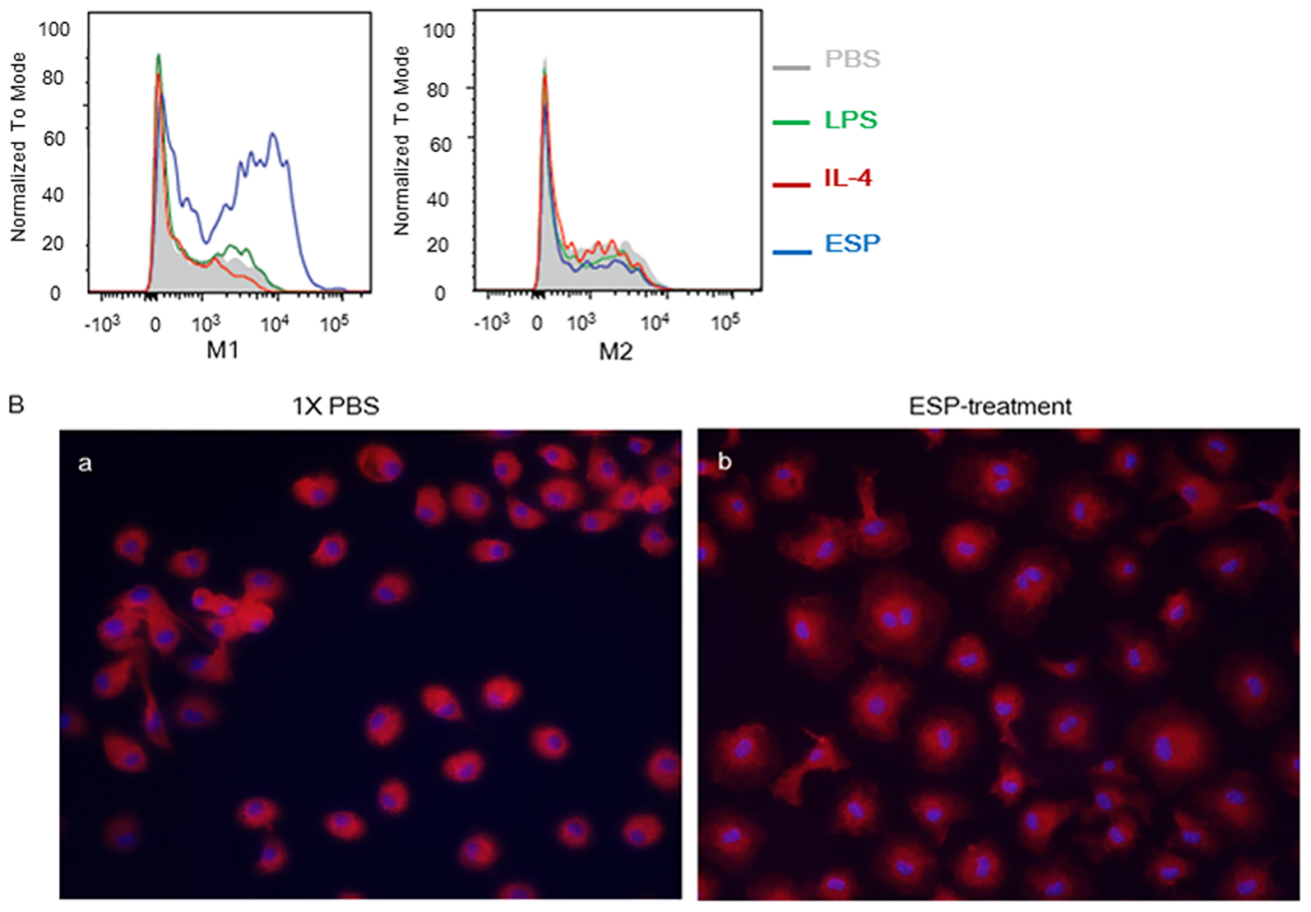
Effect of treatment with *Clonorchis sinensis* excretory/secretory proteins (ESPs) on hepatic macrophages. (A) Flow cytometry histograms of hepatic macrophages (Kupffer cells) purified from uninfected mice and stimulated *in vitro* with either PBS, 100 ng/ml LPS, 10 ng/ml IL-4, or 10 μg/ml *C*. *sinensis* ESPs. (B) Fluorescence microscopy images of normal hepatic macrophages treated for 48 h with (a) PBS or (b) 10 μg/mL *C*. *sinensis* ESPs, stained with an F4/80-specific antibody (200×).

Compared with that in controls, ESP stimulation significantly increased the proportion of M1 macrophages but not M2 macrophages (Fig 1A). Notably, the macrophages treated with 10 μg/ml *C*. *sinensis* ESPs exhibited both an increase in cell size and a “spiked” cell shape, characteristic of activated macrophages (Fig 1B).

### *C*. *sinensis* ESPs promoted cytokine production in hepatic macrophages

**Fig 2:**
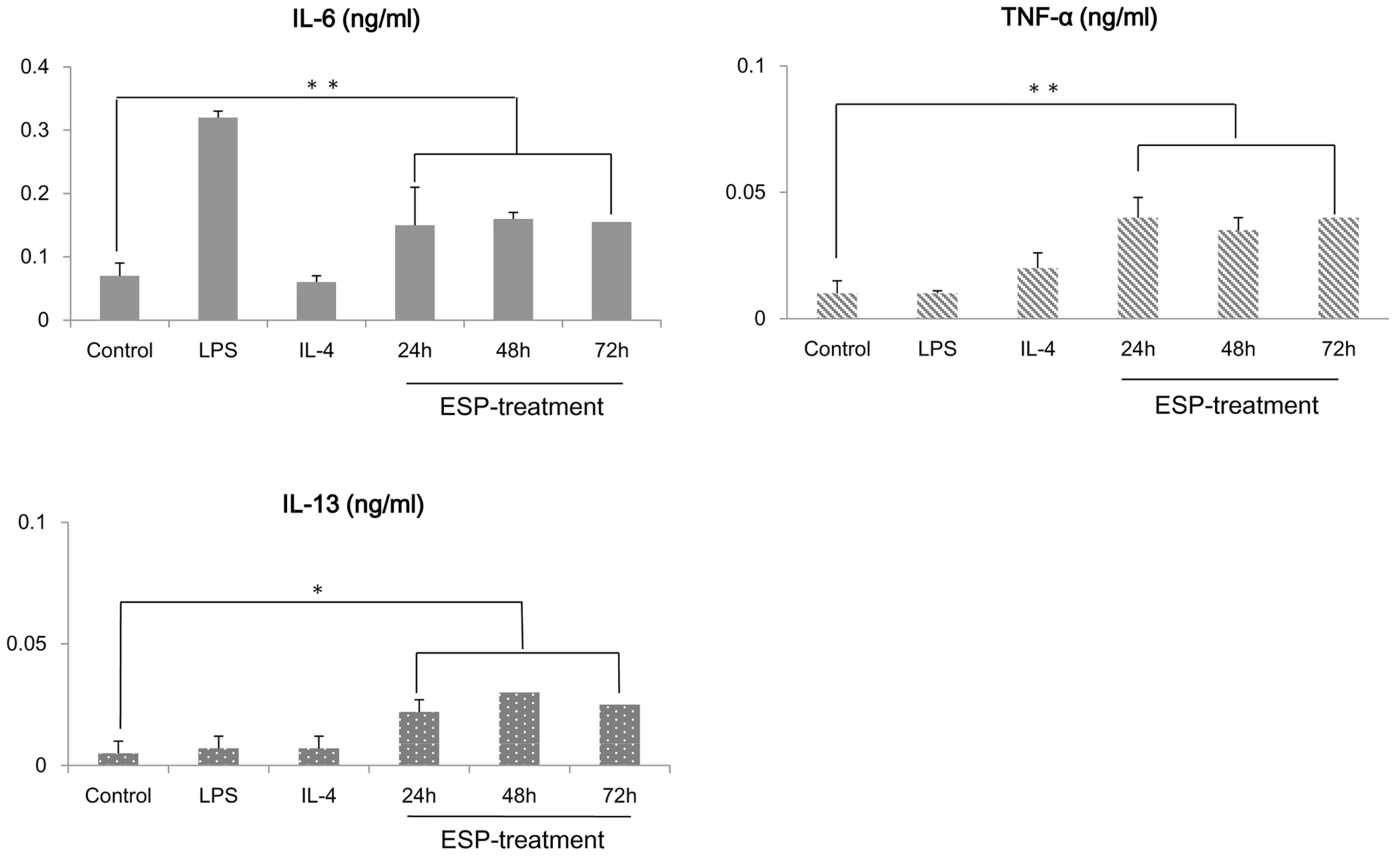
Cytokine profiles of hepatic macrophages treated with *Clonorchis sinensis* ESPs. Levels of IL-6, TNF-α, and IL-13 secreted by hepatic macrophages isolated from uninfected mice, as determined by ELISA, following treatment with either PBS (control), 100 ng/ml LPS, 10 ng/ml IL-4, or 10 μg/ml ESPs. The data are presented as the means ± standard errors of the means (SEMs) of the results obtained from at least three independent experiments. *p < 0.05, **p < 0.01 (*t*-test).

Hepatic macrophages from uninfected mice produced TNF-α, IL-6, and IL-13 in response to ESP stimulation. In particular, the stimulated cells secreted significantly more amount of the pro-inflammatory cytokines TNF-α and IL-6 than the control cell population (Fig 2).

### Histopathologic findings from *C*. *sinensis*-infected mice liver

**Fig 3:**
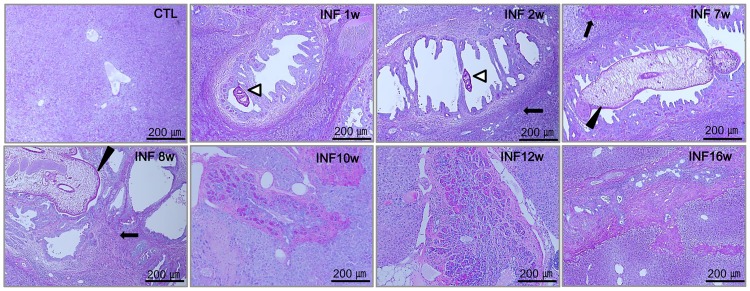
Periodic acid-Schiff (PAS) staining of mucin in the livers of *Clonorchis sinensis*-infected mice. (A scale bar = 200 *μ*m).

*C*. *sinensi*s-infected mice showed obvious infiltration of inflammatory cells and fibrocystic accumulation around intrahepatic bile ducts (Fig 3). Increasing mucin deposits within the liver were observed along with the development of fibrosis and cirrhosis. The morphological changes that occurred during clonorchiasis in mice are shown in Fig 3. Notably, hyperplasia of the bile duct epithelium was observed during early infection (Fig 3). At 1–2 weeks post-infection, there was a marked increase in the number of mucin-positive cells, mainly at the cholangiocytes. A juvenile worm arrowheads) was observed in the bile ducts, along with moderate periductal fibrosis (arrow) and bile duct proliferation. Mucin staining was strongly positive at the chronic stage. An adult worm (black arrowhead) was observed in the dilated bile duct, and massive fibrosis (arrow) was visible around the bile duct (Fig 3). Additionally, acute inflammatory cell infiltration around the bile duct and portal areas, accompanied by cholangiocyte proliferation, was observed 2 weeks post-infection. At the egg-emission stage, one adult worm (black arrowhead) was apparent in a dilated bile duct, and considerable infiltration by inflammatory cells was observed in the surrounding bile ducts. Minor collagen deposition was observed, and intrahepatic bile duct epithelium was noted. Intrahepatic bile ducts gradually became more extended until 7–8 weeks post-infection (Fig 3). In contrast, inflammatory cell infiltration appeared to be attenuated inside the bile duct after the parasite was removed, whereas collagen deposition and mucin expression were observed around the bile ducts of the livers of infected mice (Fig 3). These results also indicated that there was a positive correlation between the repaired bile ducts and mucin secretion in cholangiocytes.

### *C*. *sinensis* infection induced morphological changes in hepatic macrophages

**Fig 4:**
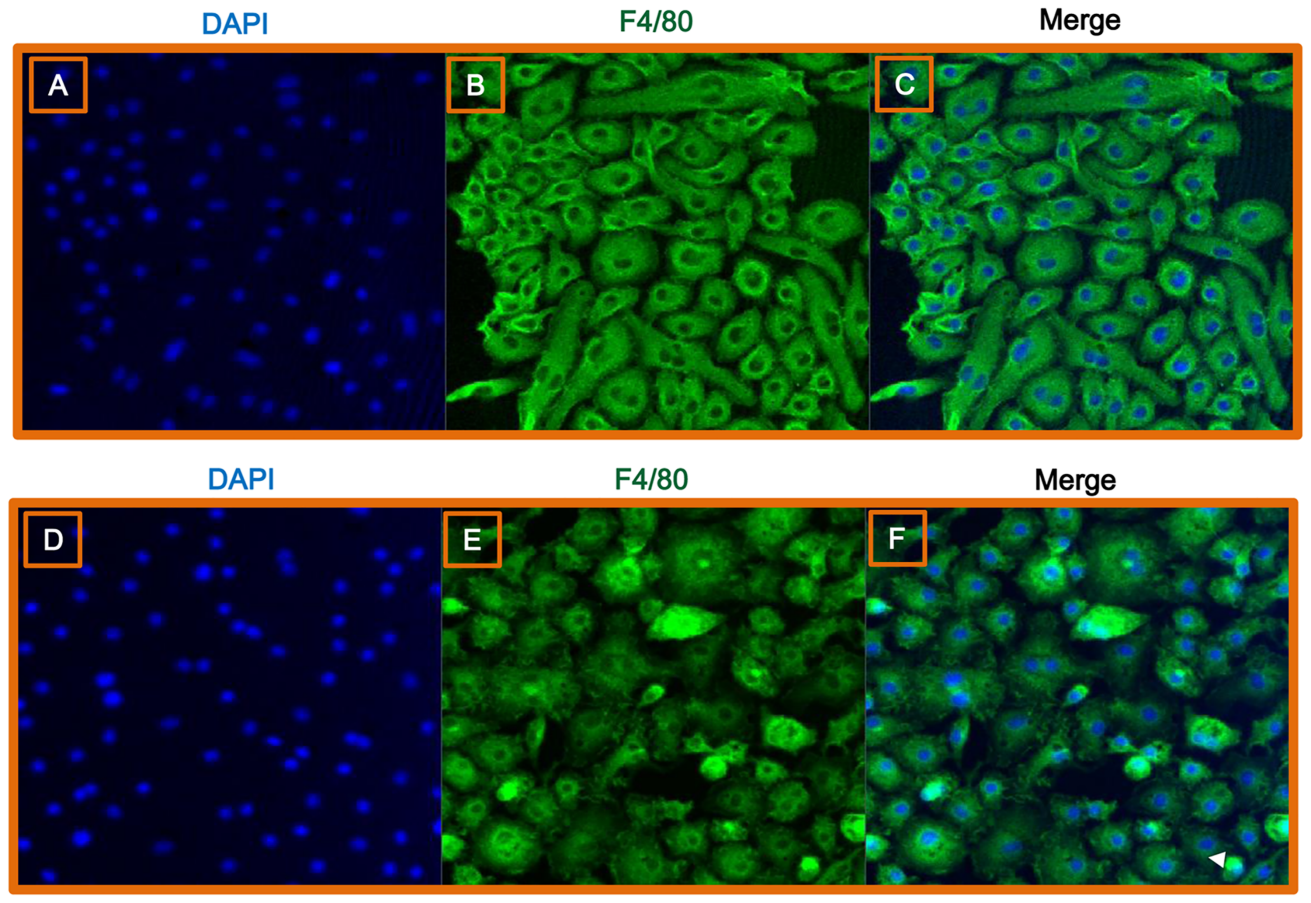
Immunofluorescent characterization of hepatic macrophages following *C*. *sinensis* infection. Fluorescence images of hepatic macrophages harvested from uninfected (A–C) and *C*. *sinensis*-infected mice (D–F). Cells were stained with DAPI (blue; A, D) or an F4/80-specific antibody (green; B, E). Cells were observed by confocal microscopy at a magnification of 200×. (C, F) Merged images.

Consistent with our *in vitro* analyses, hepatic macrophages isolated from mice infected with *C*. *sinensis* exhibited the characteristic spindle shape of activated macrophages (Fig 4).

#### Localization of hepatic macrophages and inflammatory responses

**Fig 5:**
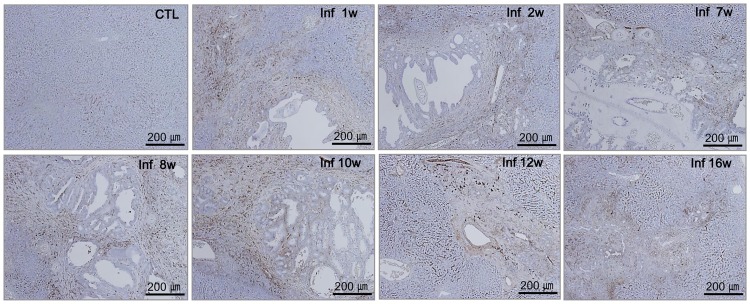
Localization of hepatic macrophages in the livers of *C*. *sinensis*-infected mice. Hepatic macrophages were detected in the liver tissues of normal (CTL) mice and mice infected with *C*. *sinensis*, using an anti-F4/80 monoclonal antibody (golden brown, A scale bar = 200 *μ*m).

The time course of hepatic macrophage localization correlated with host pathology in mice. There was strong staining of the recruited hepatic macrophages in direct contact with the flukes as well as in the surrounding area, and the presence of hepatic macrophages was associated with heavy inflammatory cell infiltration, particularly of mononuclear cells (Fig 5). During early infection, hepatic macrophages surrounded the bile ducts and veins. Additionally, the livers of cirrhotic stage mice exhibited infiltration of hepatic macrophages around the portal areas and dilated bile ducts, as well as tissue fibrosis (Fig 5). Notably, there was a marked decrease in the number of recruited hepatic macrophages after worm clearance, and an increase in recruited-macrophage count at areas of tissue remodeling during the cirrhotic stage of infection (Fig 5).

### Changes in hepatic M1 and M2 macrophage proportions during *C*. *sinensis* infection

**Fig 6:**
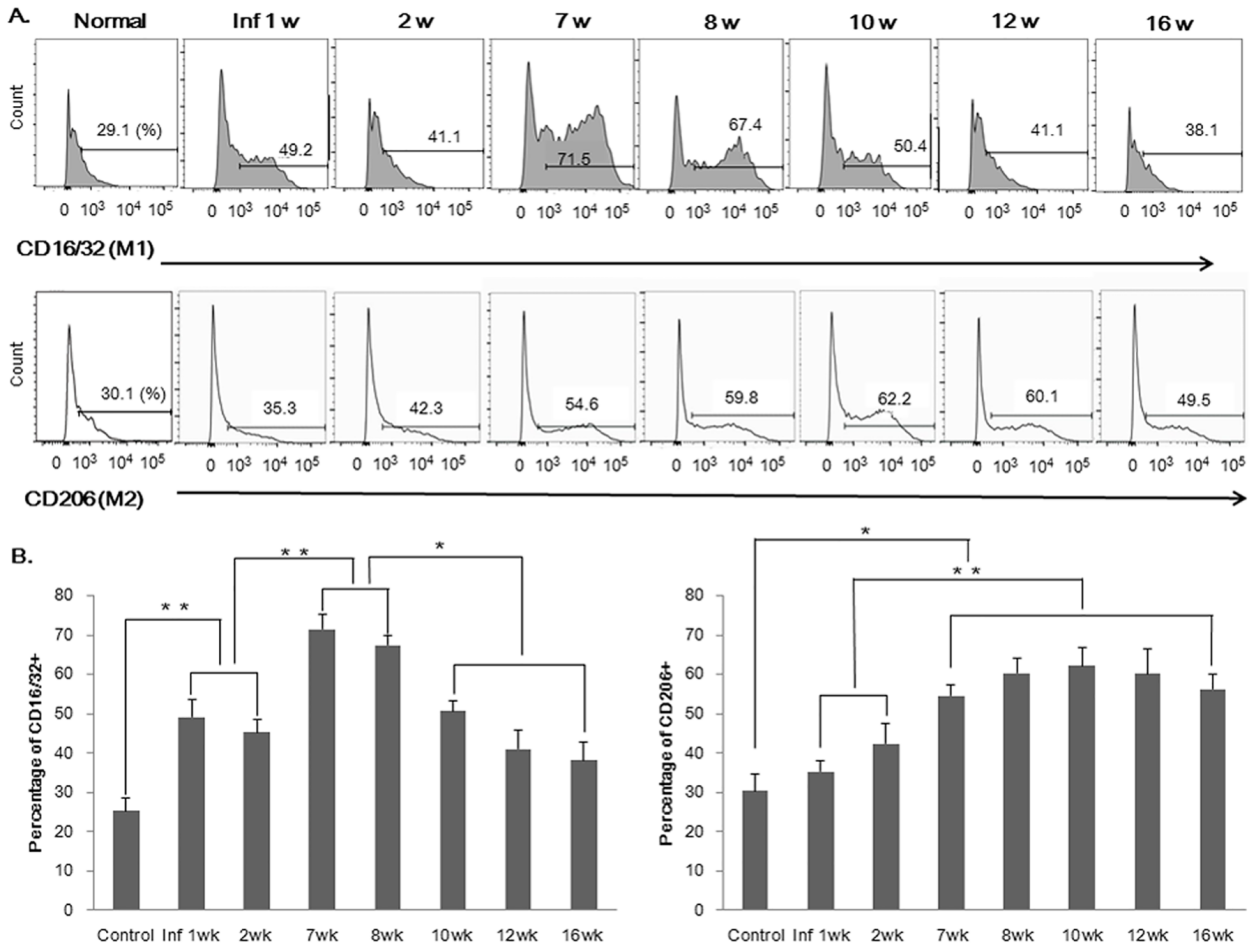
Changes in the percentages of M1 and M2 hepatic macrophages during *C*. *sinensis* infection. (A) M1 and M2 macrophages were counted by flow cytometry. (B) Graphs show the percentages of M1 and M2 macrophages within the livers of control and infected mice at each time point. *p < 0.05, **p < 0.01 (t-test).

As early as 1 week postinfection, an increase in the relative proportion of hepatic M1 macrophages was observed within the livers of infected mice; the percentage of M1 macrophages in infected mice then increased steadily until the egg-production stage, but decreased sharply at the cirrhotic stage. In contrast, the percentage of M2 macrophages decreased sharply at the beginning of the egg production stage and then peaked during the chronic stage (Fig 6).

### Expression of M1- and M2-specific enzymes in hepatic macrophages during *C*. *sinensis* infection

**Fig 7:**
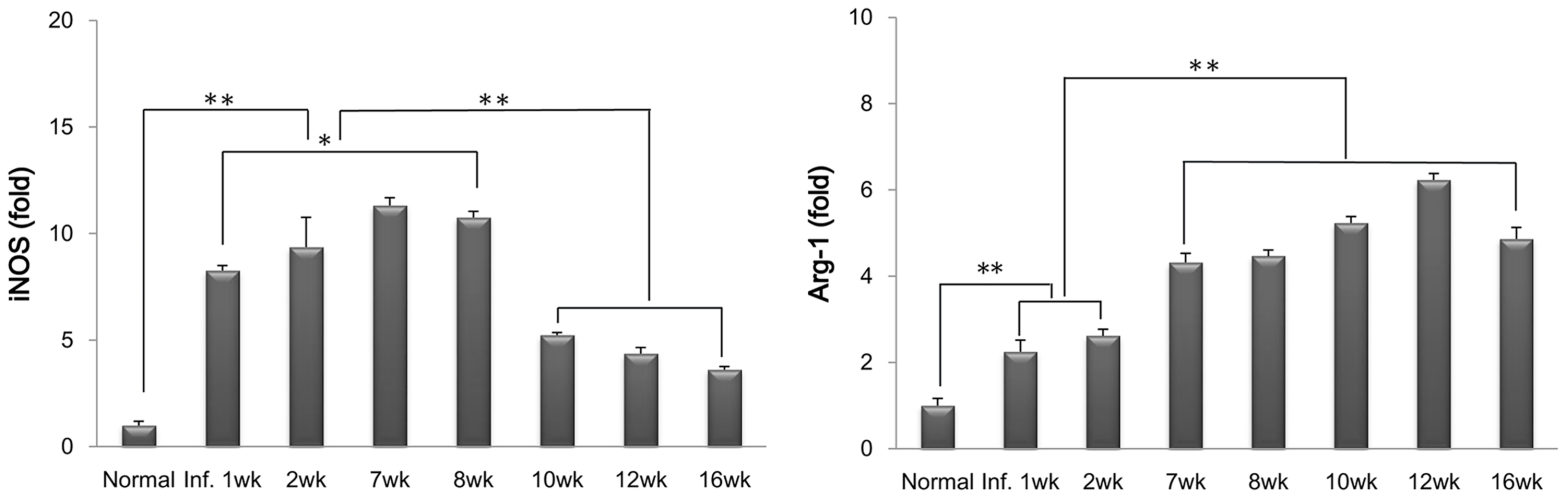
Dynamics of M1 and M2 macrophage-specific enzyme expression during *C*. *sinensis* infection. The mRNA expression levels of iNOS (*Nos2*; M1-specific) and Arg-1 (*Arg1*; M2-specific) were evaluated by real-time PCR.*p < 0.05, **p < 0.01.

As shown in Fig 7, expression of the M1-specific enzyme iNOS began to increase 1 week after infection, and increased steadily until the egg-production stage. However, its expression was significantly reduced at the fibrotic and cirrhotic stage. Conversely, the expression of Arg-1, an M2-specific enzyme, increased from 7 weeks after infection and was markedly upregulated 10–12 weeks postinfection. Interestingly, iNOS was highly upregulated at both the migration and egg-emission stages, whereas Arg-1 was strongly upregulated after the egg-migration stage.

### M1 and M2 macrophage-related chemokine and cytokine expression in hepatic macrophages in *C*. *sinensis*-infected mice

**Fig 8:**
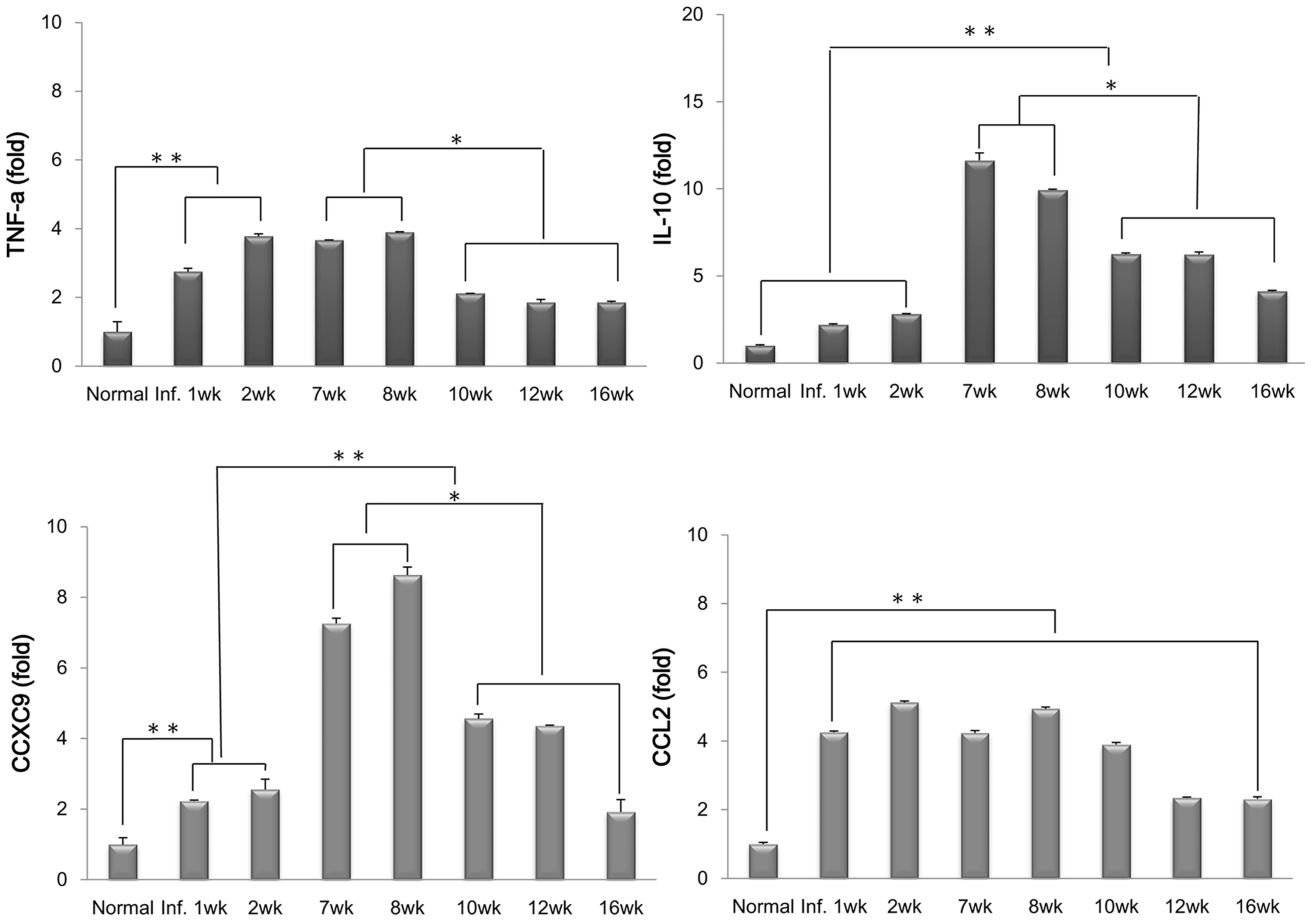
Dynamics of the expression of M1 (CD16/32-positive) and M2 (CD206-positive) macrophage-related chemokines and cytokines in hepatic macrophages during *C*. *sinensis* infection. The mRNA expression levels of CXCL9, CCL2, TNF-α, and IL-10 were evaluated by real-time PCR. *p < 0.05, **p < 0.01.

There was an increase in the expression of M1-related chemokines and CXCL9 at the peak of the egg-release stage. Additionally, a marked increase in the expression of the M2-related chemokine CCL2 was observed 1 week after infection, and expression of this factor was maintained at a high level until week 16 (Fig 8). In addition, the M1-related cytokine TNF-α was highly upregulated during acute infection, whereas IL-10 expression increased at the peak of the egg emission stage.

## Discussion

*C*. *sinensis* infection is known to primarily activate humoral immunity and Th2-type immune responses, but some studies have also described the activation of cellular immunity, particularly at the site of infection [1, 4, 21–24]. In this study, we describe for the first time, hepatic macrophage polarization in mice liver following *C*. *sinensis* infection, as well as the secreted factors that could be involved in the immune response. We show that the polarization of hepatic macrophages shifts dynamically during the different stages of *C*. *sinensis* infection. *C*. *sinensis* ESPs favored the generation of M1-type hepatic macrophages *in vitro*. In addition, during the early stage of infection, *C*. *sinensis* induced morphological changes in murine hepatic macrophages, which were functionally polarized into M1. On the other hand, M2 hepatic macrophages were increased in number during the egg-emission stage of infection, with this increase accelerating during the fibrotic and cirrhotic stage of infection.

As components of the host immune system, macrophages regulate tissue generation, help to maintain organ function, regulate metabolic mechanisms, and promote inflammatory responses [19]. This range of responses is possible because macrophage activation is dynamic, and they are able to change and adapt in response to changes in the local environment [18, 20].

Resident hepatic macrophages, known as Kupffer cells, account for 80–90% all of tissue macrophages throughout the body [14]. Notably, *C*. *sinensis* ESP secretion results from the direct interaction between *C*. *sinensis* antigens and host cholangiocytes in the bile duct. Such interactions between *C*. *sinensis* and host cells, including hepatic macrophages, drive specific immune responses in the liver, demonstrating the importance of macrophages in host defense mechanisms.

Infection with the parasite *T*. *spiralis* reportedly induces a shift towards proliferative M1 macrophages during the early stages, with M2 macrophages becoming more prevalent once the parasite progresses to the larval stage and forms cysts in muscle tissue [25]. Several studies have suggested that M1 macrophages are capable of killing schistosomula by producing nitric oxide [19–20], which plays a role in preventing the progression of fibrosis. Although our results show that M1-related factors, such as iNOS, TNF-α, and CXCL9, were observed when the M1 ratio peaked during the egg-emission stage, we were unable to determine whether adult worms were expelled from the bile ducts.

In contrast, M2 macrophages are thought to contribute to schistosome-induced fibrosis via the metabolism of Arg-1 [18–20]. Indeed, M2-specific enzymes, such as Arg-1, and M2-related chemokine activation of the CCL2-CCR2 axis are associated with monocyte infiltration in patients with chronic liver disease and fibrogenesis [14, 15]. In a mouse model of inflammatory bowel disease, peritoneal M2 macrophages induced by *T*. *spiralis* suppressed the production of pro-inflammatory cytokines such as TNF-α and IL-6, and consequently reduced the severity of inflammation [25, 26].

In the present study, Arg-1 levels were maintained throughout the infection, with the highest level occurring at the fibrotic and cirrhotic stage. A previous study showed that the worm recovery rate decreased with prolonged infection, and that worms were expelled from mice over time [23]. Another study reported that the number of Kupffer cells, acting as antigen presenting cells, is increased 70-fold in the early stages of clonorchiasis when compared to that in controls [24]. However, until now, there has been no convincing evidence demonstrating that M2 macrophages are involved in the expulsion of worms from mice. Our data showed very little evidence of the presence of worms in the bile ducts, using histological methods more than 10 weeks after infection, and the increase in the proportion of M2 macrophages, involved in tissue regeneration and the remodeling of the surrounding bile duct, at these stages was consistent with a strong immune response. Furthermore, we detected the expression of mucin at the site of worm expulsion. Mucin was previously shown to be overexpressed in the intestine during an immune response to hookworm infection and is known to contribute to the expulsion of hookworms [27]. Consistent with this, we observed convincing collagen deposition and mucin expression in the liver during the fibrotic and cirrhotic stage of *C*. *sinensis* infection. These results suggest a correlation between the repair of bile ducts following worm expulsion and mucin secretion in cholangiocytes, although the connection between hepatic macrophages and mucin secretion following *C*. *sinensis* infection is currently unclear. In future studies, it will be interesting to examine the relationships between the phenotypes of polarized hepatic macrophages and mucin-secreting cells. Furthermore, our results indicate that M2 hepatic macrophages modulate fibrosis and the tissue repair processes that are necessary to transform bile ducts from the thickened, dilated morphology caused by *C*. *sinensis* infection to a narrower morphology. Further studies are needed to determine whether immunomodulation depends on specific *C*. *sinensis* ESP molecules *in vitro* or whether the elimination of murine M2 macrophages *in vivo* affects worm expulsion or survival.

In summary, our results suggest that the modulation of hepatic macrophage polarization by *C*. *sinensis* infection may serve as a potential mechanism for both parasite immune escape and host tissue repair. Furthermore, the dynamic polarization of hepatic macrophages as infection progresses corresponds to histological lesions within liver tissue. Hepatic macrophages thus play an important role in local immunity during *C*. *sinensis* infection.
